# Effect of Potent P2Y_12_ Inhibitors on Ventricular Arrhythmias and Cardiac Dysfunction in Coronary Artery Disease: A Systematic Review and Meta-Analysis

**DOI:** 10.1155/2018/8572740

**Published:** 2018-12-17

**Authors:** Chunmei Wang, Guanqi Zhao, Xiao Wang, Shaoping Nie

**Affiliations:** Emergency & Critical Care Center, Beijing Anzhen Hospital, Capital Medical University, Beijing, China

## Abstract

**Background:**

Previous studies have shown that P2Y_12_ receptor inhibitors might prevent ventricular arrhythmias and cardiac dysfunction in patients with coronary artery disease. However, few studies have focused on comparison of the efficacy of novel oral potent P2Y_12_ receptor inhibitors with clopidogrel on these outcomes.

**Methods and Results:**

We performed a systematic review and meta-analysis of randomized controlled trials (RCTs) that were published in electronic databases of MEDLINE, EMBASE, Cochrane Central Register of Clinical Trials, and ClinicalTrials.gov before June 20, 2018. We compared the effect of prasugrel and ticagrelor with clopidogrel on outcomes of ventricular tachycardia (VT), ventricular fibrillation (VF), heart failure (HF), and cardiogenic shock (CS). Data were combined using both the fixed-effects models and the random-effects models, and the heterogeneity was assessed with the *I*^2^ statistic. Nine RCTs (6 with prasugrel and 3 with ticagrelor) with 45,227 patients were included. Patients receiving prasugrel were associated with a lower risk of combined VT and VF (rate ratio [RR]: 0.72, 95% confidence interval [CI]: 95% CI: 0.52-0.99,* p*=0.043), as well as combined HF and CS (RR: 0.81, 95% CI: 0.70-0.94,* p*=0.005), compared with clopidogrel. Patients receiving ticagrelor were also associated with a reduced risk of VT and VF (RR: 0.85, 95% CI: 0.72-1.02,* p*=0.077), although without statistical significance, but not of HF and CS (RR: 0.96, 95% CI: 0.81-1.13,* p*=0.620).

**Conclusions:**

This meta-analysis of RCTs shows that, compared with clopidogrel, novel oral P2Y_12_ inhibitors, especially prasugrel, might have better effect on improving ventricular rhythm and cardiac function.

## 1. Introduction

For decades, dual antiplatelet therapy with aspirin and P2Y purinoceptor 12 (P2Y_12_)-receptor inhibitor of clopidogrel has remained the cornerstone of treatment for patients with acute coronary syndrome (ACS). The novel oral P2Y_12_ receptor inhibitors of prasugrel and ticagrelor, approved by the FDA for clinical use in 2009 and 2011, have also been recommended as the first-line therapy for patients with ACS in the guideline based on their rapid onset of action and potent effects on inhibition of platelet aggregation [1, 2], as well as a better effect on lowering risk of all-cause death and major adverse cardiovascular events (MACE) [3–8]. However, whether these two novel oral P2Y_12_ receptor inhibitors, compared with clopidogrel, have better effect on improving heart rhythm and heart function is unclear.

Ventricular arrhythmias and cardiac dysfunction are severe complications that can significantly increase the risk of death and rehospitalization for patients with coronary artery disease (CAD), especially for those with ACS [5]. Studies have suggested that antiplatelet agents might have an effect on improving ventricular rhythm and cardiac function by reducing the frequency of coronary thrombotic occlusions [9, 10]. However, few studies have focused on comparison of the effect of these novel oral P2Y_12_ inhibitors with clopidogrel on these complications.

This meta-analysis reviewed relevant randomized controlled trials (RCTs) to compare the effects of prasugrel and ticagrelor on ventricular tachycardia (VT), ventricular fibrillation (VF), heart failure (HF), and cardiogenic shock (CS), with those of clopidogrel. We aimed to determine whether prasugrel or ticagrelor may improve prognosis by reducing ventricular arrhythmias and cardiac dysfunction.

## 2. Materials and Methods

### 2.1. Data Sources and Search Strategy

We conducted a meta-analysis according to Preferred Reporting Items for Systematic Reviews and Meta-Analyses (PRISMA) [25]. We searched the electronic databases of MEDLINE, EMBASE, Cochrane Central Register of Clinical Trials, and ClinicalTrials.gov with no language restriction to identify all published or registered RCTs. All of these searches were conducted by 2 independent researchers (C.M.W. and G.Q.Z.) before June 20, 2018. The following terms were used: “coronary artery disease” OR “coronary heart disease” OR “acute coronary syndrome” OR “acute myocardial infraction” OR “ST-elevation myocardial infraction” OR “non-ST-elevation myocardial infraction” OR “unstable angina” OR “non-ST-elevation acute coronary syndromes” OR “stable angina” OR “percutaneous coronary intervention” and “ticagrelor” OR “prasugrel” and “clopidogrel” and “heart failure” OR “cardiogenic shock” OR “ventricular tachycardia” OR “ventricular fibrillation” OR “ventricular arrhythmias” and their synonyms or variations. Reference lists of selected studies, relevant articles, and related systematic reviews were manually reviewed for potential retrieved studies.

### 2.2. Study Selection and Data Extraction

**Figure 1:**
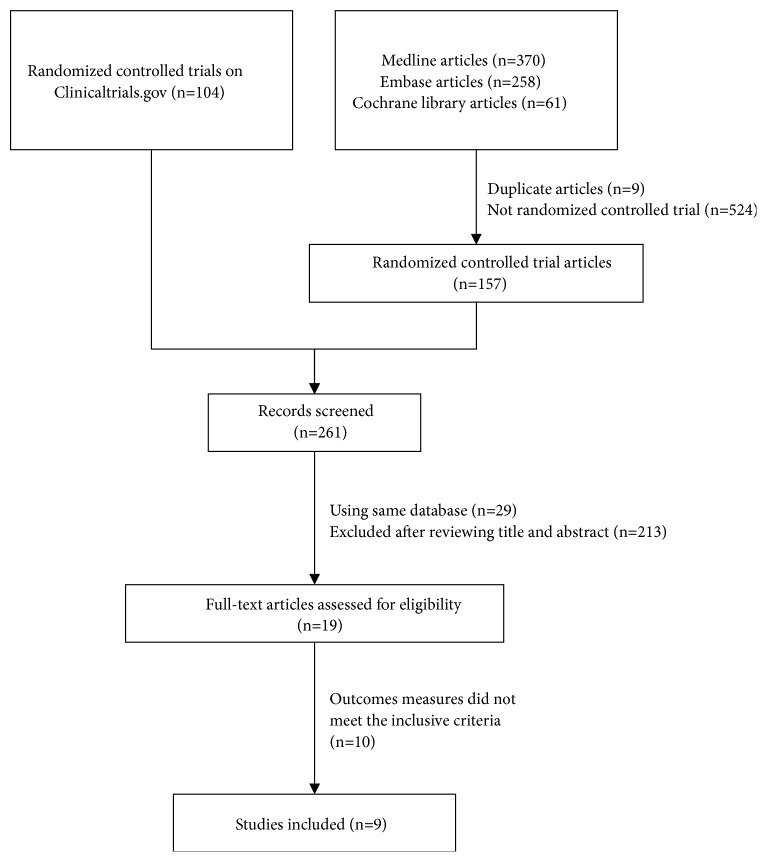
Review process for inclusion/exclusion of studies.

RCTs that compared the outcomes of prasugrel or ticagrelor with clopidogrel in adults (≥18 years) with all forms of CAD were included to screen whether they reported at least one of the following outcomes: VT, VF, HF, and CS. Studies not reporting the clinical outcomes of interest were excluded. Meeting abstracts and studies that only reported antiplatelet effects of these agents were also excluded. The details of the study selection are described in Figure 1.

Data extraction was performed independently by 2 researchers (C.M.W. and G.Q.Z.) with prepared standardized data forms. Divergent assessments were resolved by discussion with a third researcher (W.X.). Study information was recorded as follows: year of the study, study intervention, number of patients, characteristics of study population, primary endpoints and follow-up duration. Detailed patients' characteristics (age, sex, proportion of patients with ACS and PCI, medical history) and crude events of VT, VF, HF, or CS during follow-up were also reported.

### 2.3. Outcomes

The outcomes of this study included VT, VF, HF, and CS, either reported in published journal articles or posted on ClinicalTrial.gov. HF was considered present if there was any reported congestive HF, acute or chronic HF. VT was considered present if sustained or nonsustained VT was reported. Other outcomes were defined according to the definitions in the respective studies.

### 2.4. Quality Assessment

The quality of RCTs was assessed using methods recommended by the Cochrane Collaboration on the basis of the following components: random sequence generation; allocation concealment; blinding of participants, personnel, and outcome assessors; incomplete outcome data; selective outcome reporting; and other sources of bias [26] (Supplementary Figure 1).

### 2.5. Statistical Analysis

The meta-analysis was performed using STATA 12.0 (StataCorp LLC, Texas, USA). Rate ratios (RRs) and 95% confidence intervals (CIs) were used as summary estimates. The pooled RRs of prasugrel and ticagrelor versus clopidogrel were calculated with both the fixed-effects model and random-effects model as heterogeneity may still exist even if* I*^2^ <50.* I*^2^ >50% suggested that heterogeneity between trials was of statistical significance. A 2-sided* p* value <0.05 was considered statistically significant. Sensitivity analysis was performed by excluding trials which were examined to be main sources of heterogeneity. Funnel diagrams of the included studies are shown in Supplementary Figure 2 to estimate the publication bias. Quality assessment was performed with Review Manager 5.3 (The Nordic Cochrane Centre, The Cochrane Collaboration, Denmark).

## 3. Results and Discussion

### 3.1. Included Studies

Based on initial research criteria, 793 publications from MEDLINE, EMBASE, Cochrane Central Register of Clinical Trials, and ClinicalTrials.gov were identified. After duplicates and non-RCTs were excluded, 261 potentially relevant publications were included for further screening and 19 publications that fulfilled the eligibility criteria were included for full text review. Nine of these publications with interesting outcomes for this study were eventually included in the present meta-analysis [1, 2, 11–17].

The characteristics of each study and detailed characteristics of patients in each study are shown in Tables 1 and 2. There were some differences among the included studies regarding the study designs and patients' characteristics. Because there were differences between ticagrelor and prasugrel, we compared the efficacy of ticagrelor and prasugrel with clopidogrel, respectively. Because not all studies provided all outcomes of interest, we summarized the outcomes of each study (Table 3). There was a total of 45,227 patients (23,102 in the potent P2Y_12_ inhibitor arm and 22,125 in the clopidogrel arm). In the nine included studies, six studies compared prasugrel with clopidogrel in 24,846 patients and three studies compared ticagrelor with clopidogrel in 20,381 patients.

### 3.2. Analysis of Ventricular Arrhythmias

**Figure 2:**
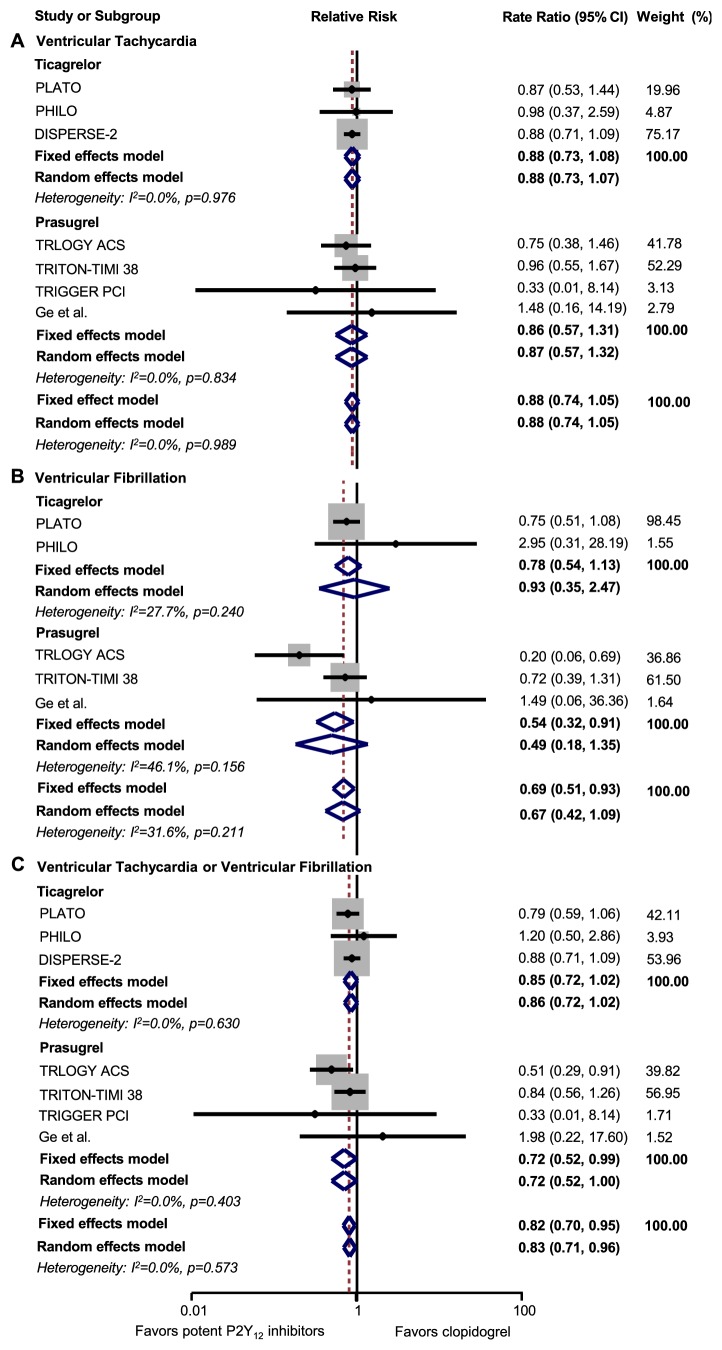
Forest plot for ventricular tachycardia, ventricular fibrillation, and pooled data of ventricular arrhythmias. A, ventricular tachycardia; B, ventricular fibrillation; C, ventricular tachycardia or ventricular fibrillation. CI: confidence interval.

Four and three studies compared the effects of prasugrel [2, 14–16, 21–23] and ticagrelor [1, 11, 12, 18, 19] with clopidogrel on VT, respectively. Compared with clopidogrel, prasugrel (RR: 0.86, 95% CI: 0.57-1.31,* p=*0.494*; I*^2^=0) and ticagrelor (RR: 0.88, 95% CI: 0.73-1.08,* p=*0.220*; I*^2^=0) were not significantly associated with reduced risk of VT (Figure 2).

Three studies compared the effects of prasugrel and clopidogrel on VF [2, 14, 15, 21–23]. Prasugrel was associated with a 46% reduced risk of VF compared with clopidogrel (RR: 0.54, 95% CI: 0.32-0.91,* p=*0.020*; I*^2^=46.1%) (Figure 2). Two studies compared the effect of ticagrelor and clopidogrel on VF [1, 12, 18, 19], but we did not observe a significantly reduced risk of VF in ticagrelor (RR: 0.78, 95% CI: 0.54-1.13,* p=*0.184*; I*^2^=27.7%) (Figure 2).

When we pooled VT and VF, both being manifestation of ventricular arrhythmias, a 28% reduced risk was observed in prasugrel (RR: 0.72, 95% CI: 0.52-0.99,* p=*0.043*; I*^2^=0) and a 15% reduced risk with ticagrelor (RR: 0.85, 95% CI: 0.72-1.02,* p=*0.077*; I*^2^=0) although without statistical significance, compared with clopidogrel (Figure 2).

### 3.3. Analysis of Cardiac Dysfunction

**Figure 3:**
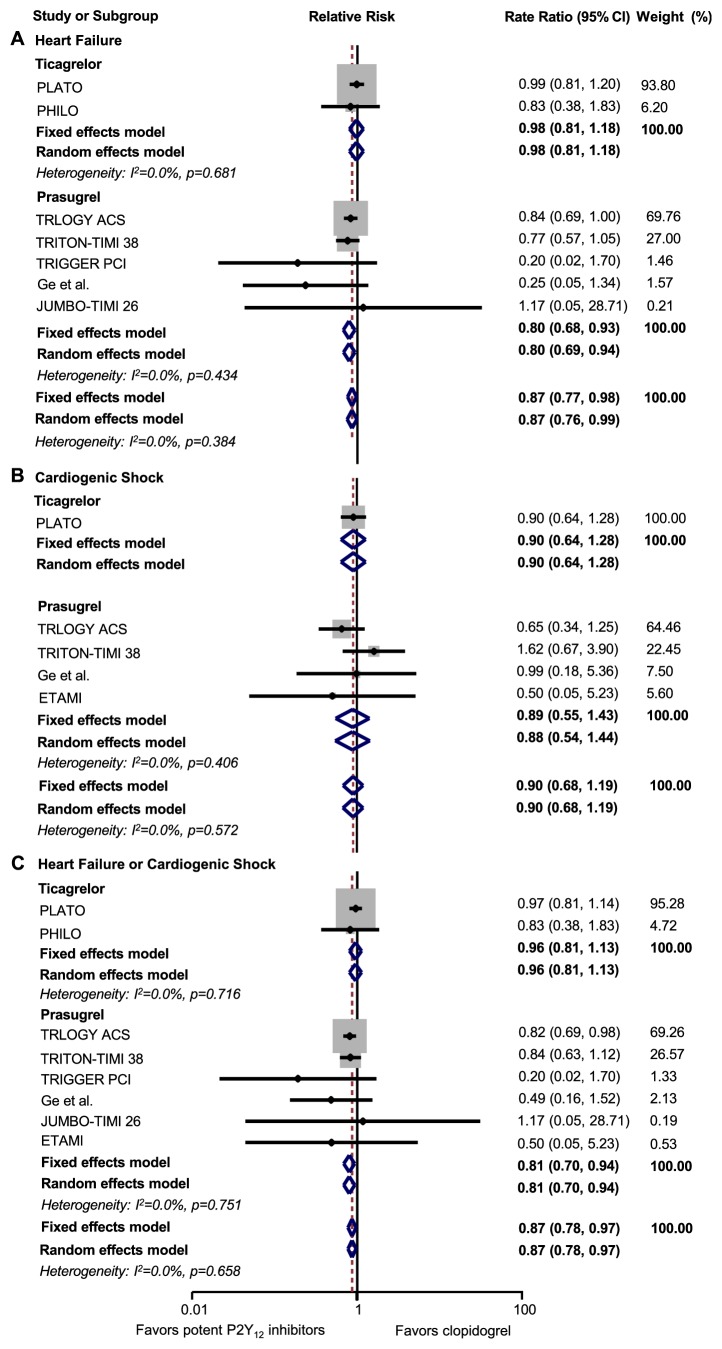
Forest plot for heart failure, cardiogenic shock, and pooled data of cardiac dysfunction. A, heart failure; B, cardiogenic shock; C, heart failure or cardiogenic shock. CI: confidence interval.

Five studies compared the effects of prasugrel and clopidogrel on HF [2, 13–16, 20–24]. Prasugrel was associated with a 20% reduced risk of HF compared with clopidogrel (RR: 0.80, 95% CI: 0.68-0.93,* p=*0.005*; I*^2^=0) (Figure 3). Two studies compared the effects of ticagrelor and clopidogrel on HF [1, 12, 18, 19]. We did not observe a reduced risk of HF in ticagrelor compared with clopidogrel (RR: 0.98, 95% CI: 0.81-1.18,* p=*0.801*; I*^2^=0) (Figure 3).

Four studies compared the effects of prasugrel and clopidogrel on CS [2, 14, 15, 17, 21–23]. Only the Platelet Inhibition and Patient Outcomes (PLATO) trial [1, 18] reported the incidence of CS in patients who took ticagrelor and clopidogrel. However, a reduced risk of CS was not observed with both prasugrel (RR: 0.98, 95% CI: 0.68-1.19,* p=*0.617*; I*^2^=0) and ticagrelor (RR: 0.90, 95% CI: 0.64-1.28,* p=*0.574) compared with clopidogrel (Figure 3).

When we pooled HF and CS, both being manifestation of cardiac dysfunction, patients with prasugrel had 19% lower risk (RR: 0.81, 95% CI: 0.70-0.94,* p=*0.005*; I*^2^=0), but not with ticagrelor (RR: 0.96, 95% CI: 0.81-1.13,* p=*0.620*; I*^2^=0), compared with clopidogrel (Figure 3).

### 3.4. Sensitivity Analysis

Among the studies, the proportions of patients with ACS in the testing platelet reactivity in patients undergoing elective stent placement on clopidogrel to guide alternative therapy with prasugrel (TRIGGER PCI) study [16] and joint utilization of medications to block platelets optimally thrombolysis in myocardial infarction 26 (JUMBO-TIMI 26) study [13] were significantly lower than those in other studies. Therefore, we performed sensitivity analyses of patients with ACS by excluding studies that showed similar rates of all the outcomes tested. We found that the RR of VT was 0.89 (95% CI: 0.66-1.21,* p*=0.967*; I*^2^=0%) and the RR of VF was 0.69 (95% CI: 0.51-0.93, p=0.014*; I*^2^=31.6%), that of VT and VF was 0.78 (95% CI: 0.63-0.96,* p=*0.022*; I*^2^=0%), the RR of HF was 0.87 (95% CI: 0.77-0.98,* p=*0.027*; I*^2^=11.3%) and the RR of CS was 0.90 (95% CI: 0.68-1.19,* p*=0.454*; I*^2^=0%), and that of HF and CS was 0.88 (95% CI: 0.78-0.98,* p=*0.020*; I*^2^=0%). The incidence of VT in the dose confirmation study assessing antiplatelet effects of AZD6140 versus clopidogrel in non-ST-segment elevation myocardial infarction-2 (DISPERSE-2) [11] was much higher than that in the other studies because of the definition of VT in this study. Therefore, sensitivity analysis that excluded this study showed that the RR of VT was 0.88 (95% CI: 0.65-1.19,* p=*0.405*; I*^2^=0%) and that of VT and VF was 0.78 (0.63-1.96,* p=*0.019*; I*^2^=0%). We also preformed sensitivity analysis by including studies with relatively large sample size (>5000) or with relatively long follow-up (>6 months). Similar results to the main result were observed (Supplementary Figures 3, 4, 5, and 6).

### 3.5. Discussion

To the best of our knowledge, this is the first meta-analysis that performs a comparison between novel oral P2Y_12_ inhibitors and clopidogrel on outcomes of cardiac dysfunction and ventricular arrhythmias in patients with CAD.

Our meta-analysis showed that potent P2Y_12_ inhibitors, including prasugrel and ticagrelor, were associated with a lower risk of ventricular arrhythmias (although there was no significance with ticagrelor compared with clopidogrel). A lowered risk of HF and CS was observed in patients taking prasugrel. In summary, compared with clopidogrel, use of prasugrel or ticagrelor could further improve ventricular rhythm and cardiac function to some extent.

Prompt and adequate dual antiplatelet therapy is essential for patients with ACS. Myocardial ischemia caused by acute thrombosis leads to severe metabolic, electrophysiological, and structural changes in the ventricular myocardium that induce life-threatening arrhythmias and heart failure [6, 27]. These directly lead to sudden cardiac death in some situations. Acute myocardial ischemia leads to ionic imbalance, less contractile force by events that culminate in mishandling of intracellular calcium, and a reduced conduction velocity because of less functional gap junctions [28]. Additionally, currents flowing from the ischemic/reperfused zones to the nonischemic zones are also important mechanisms of ventricular arrhythmias [29]. In patients with ACS, ventricular myocardium may be ischemic, stunned, hibernating, or irrevocably injured. Ventricular remodeling after onset of ACS may cause CS by mechanical complications and HF by contractile dysfunction and derangement of cardiac structure [30]. Platelet activation by the time or after myocardial infarction also plays an important role in cardiac remodeling by its proinflammatory effects apart from prothrombotic effects [31, 32].

The trend of oral antiplatelet agents reducing the incidence of ventricular arrhythmias and dysfunction has been observed in previous studies. In the landmark second international study of infarct survival (ISIS-2), antiplatelet therapy with aspirin in patients with myocardial infarction significantly reduced the incidence of VF compared with placebo (4.3% versus 5.1%,* p*=0.022) [33]. The clopidogrel in unstable angina to prevent recurrent events (CURE) trial also showed that, compared with aspirin alone, the benefits of cardiac function were observed in aspirin in addition to clopidogrel. Clopidogrel significantly reduced the risk of heart failure (3.6% versus 4.5%,* p*=0.017) in the CURE trial [34].

As a prodrug, clopidogrel has several limitations, such as requiring hepatic conversion, low bioavailability, relatively slow onset of action, and variability in responsiveness in patients [35]. Pharmacodynamics and pharmacokinetics studies have shown that prasugrel and ticagrelor have a greater and more rapid inhibition of platelet aggregation [36, 37]. A meta-analysis of phase III/IV RCTs showed better efficacy on MACE and all-cause death of these 2 potent P2Y_12_ inhibitors compared with clopidogrel [38]. The real-world outcomes were consistent with RCTs. In the SWEDEHEART registry, post-ACS use of ticagrelor was associated with a lower risk of death and ischemic events compared with clopidogrel [39]. These new drugs could induce earlier and more complete inhibition of platelets, leading to a lower thrombus burden and platelet-induced ventricular remodeling. In the CvLPRIT study, the novel P2Y_12_ inhibitors were associated with smaller infarct size and lower microvascular obstruction incidence versus the clopidogrel for ST-segment elevation myocardial infarction [40]. This would result in a lower rate of cardiac dysfunction and ventricular arrhythmias [41]. This may partially explain why novel P2Y_12_ inhibitors have a significantly protective effect on mortality in patients with CAD. Further studies on the exact mechanisms of these inhibitors are required.

Furthermore, ticagrelor was proved to provide extra effects on myocardial protection beyond the inhibition of P2Y_12_ receptor. In vitro studies indicated that, compared with clopidogrel, ticagrelor could limit myocardial infarct size and reduce myocardial edema and reperfusion injury by adenosine-mediated effects, improving endothelial function and dampening release of inflammatory mediators [42–46]. However, limited studies were conducted to explore cardioprotective mechanism of prasugrel [47]. In a recent meta-analysis of observational and randomized studies, prasugrel seems to be equivalent or superior to ticagrelor in ACS patients undergoing PCI on the 30-day outcomes [48]. But future randomized trials are still needed to evaluate the superiority of these drugs.

### 3.6. Limitations

This meta-analysis has several limitations. First, trials included in our study had different sample sizes, hypotheses, inclusion and exclusion criteria, and duration of follow-up and varied drug doses of potent P2Y_12_ inhibitors. Therefore, there must be potential heterogeneity between studies although tests for heterogeneity were of no statistical significance. Second, this analysis was not based on the results of the main outcomes from each trial, which may not be adjudicated by clinical end point committee. In addition, the definitions of cardiac function (CS/HF) and rhythm (VT/VF) outcomes varied among trials, which resulted in a varied incidence of outcomes in each study. Especially for ventricular arrhythmias, it is unknown whether events that occurred in the index event phase were included. DISPERSE-2, which had a relatively higher incidence of VT compared with other studies, was excluded in a sensitivity analysis. The results remained almost unchanged in this sensitivity analysis. Third, as the incidences of VT and VF were very low, the net benefit of prasugrel still needs to be considered. Finally, limited original clinical studies reporting the effects of novel P2Y_12_ receptor inhibitors on cardiac rhythm and cardiac function limited the reliability of the results, especially for ticagrelor, clinical evidence of which was mostly based on PLATO studies. Therefore, more studies are still needed to explore the effect of these novel P2Y_12_ inhibitors.

## 4. Conclusions

This meta-analysis of RCTs shows that novel oral P2Y_12_ inhibitors, especially prasugrel, might have better effect on improving ventricular rhythm and cardiac function compared with clopidogrel, which, to some extent, explained the reasons for the improved prognosis of these novel oral P2Y_12_ inhibitors. However, future special studies are still needed to reevaluate these results.

## Figures and Tables

**Table 1:** Characteristics, designs, and follow-up durations of the included studies.

Name	Year	Intervention	Potent P2Y_12_ inhibitors, n	Clopidogrel, n	Characteristics of Study Population	Primary Endpoint	Follow-up
DISPERSE-2 [11]	2007	Ticagrelor vs Clopidogrel	663	327	Patients were hospitalized for NSTE-ACS within the preceding 48 hours.	Major adverse cardiac event; Major bleeding (fatal/life-threatening)	12 weeks (primary endpoint)
PLATO [1]	2009	Ticagrelor vs Clopidogrel	9333	9291	Hospitalized patients with ACS.	CV death, MI, or stroke; Major bleeding, study criteria	12 months (primary endpoint)
PHILO [12]	2015	Ticagrelor vs Clopidogrel	387	380	Patients with non-ST or ST segment elevation ACS	Major adverse cardiac event; Major bleeding	12 months (primary endpoint)
JUMBO-TIMI26 [13]	2005	Prasugrel vs Clopidogrel	651	254	Patients candidate for elective or urgent PCI with intended coronary stenting.	Major adverse cardiac event; Non-CABG TIMI Major or Minor Bleeding Events	30 days (primary endpoint)
TRITON-TIMI 38 [2]	2007	Prasugrel vs Clopidogrel	6741	6716	Patients with ACS and scheduled for PCI	CV death, nonfatal MI, or nonfatal stroke; TIMI non-CABG major bleeding	14.5 months (median)
Ge et al. [14]	2010	Prasugrel vs Clopidogrel	463	299	East or southeast Asian patients with ACS and scheduled for PCI.	ADP-Induced P2Y_12_ PRU at 4 hours and 30 days	30 days (primary endpoint)
TRILOGY ACS [15]	2012	Prasugrel vs Clopidogrel	4623	4617	Patients with ACS who were medically managed.	CV death, nonfatal MI, or nonfatal stroke; TIMI non-CABG major bleeding	17.1 months (median)
TRIGGER PCI [16]	2012	Prasugrel vs Clopidogrel	210	210	Coronary artery disease patients underwent PCI with at least one drug-eluting stent implantation.	Composite endpoint of CV death or MI	6 months (primary endpoint)
ETAMI [17]	2015	Prasugrel vs Clopidogrel	31	31	Patients with acute STEMI ≤ 12 hours and scheduled for PCI	Platelet reactivity index (PRI) 2 hours after the initiation of the therapy	30 days (clinical events)

DISPERSE-2: dose confirmation study assessing anti-platelet effects of AZD6140 vs. clopidogrel in non-ST-segment elevation myocardial infarction-2, PLATO: platelet inhibition and patient outcomes, PHILO: ticagrelor vs. clopidogrel in Japanese, Korean, and Taiwanese patients with acute coronary syndrome, JUMBO-TIMI26: joint utilization of medications to block platelets optimally-thrombolysis in myocardial infarction 26, TRITON-TIMI 38: trial to assess improvement in therapeutic outcomes by optimizing platelet inhibition with prasugrel–thrombolysis in myocardial infarction, TRILOGY ACS: the targeted platelet inhibition to clarify the optimal strategy to medically manage acute coronary syndromes, TRIGGER PCI: testing platelet reactivity in patients undergoing elective stent placement on clopidogrel to guide alternative therapy with prasugrel, ETAMI: early thienopyridine treatment to improve primary PCI in patients with acute myocardial infarction, PCI: percutaneous coronary intervention, NSTE-ACS: non-ST-segment elevation acute coronary syndrome, ACS: acute coronary syndrome, CABG: coronary artery bypass grafting, CV: cardiovascular, TIMI: thrombolysis in myocardial infarction, MI: myocardial infarction, NSTEMI: non-ST-segment elevation myocardial infarction, STEMI: ST-segment elevation myocardial infarction, GUSTO: Global use of strategies to open occluded coronary arteries, ADP: adenosine diphosphate, PRU: P2Y_12_ reaction units.

**Table 2:** Patients' characteristics of included RCTs.

Study	Age (years)*∗*	Male (%)	ACS (%)	PCI (%)	Smoker (%)	Diabetes mellitus (%)	Hypertension (%)	Prior MI (%)
DISPERSE-2 [11]	63.0±11.6	63.7	100.0	42.0	N/A	24.8	N/A	26.0
PLATO [1]	62.2 (53.0, 70.0)	74.8	99.8	76.8	35.9	23.2	65.4	17.0
PHILO [12]	67.0±11.0	76.4	100.0	84.6	38.5	34.7	74.3	8.0
JUMBO-TIMI26 [13]	59.2±9.03	77.0	40.0	100.0	28.4	25.0	N/A	N/A
TRITON-TIMI 38 [2]	60.9±11.3	74.1	100.0	100.0	38.0	23.0	64.0	18.0
Ge et al. [14]	60.8±11.1	74.8	100.0	100.0	N/A	N/A	N/A	N/A
TRILOGY ACS [15]	65.7±11.0	60.9	100.0	0	39.9	37.9	82.0	42.8
TRIGGER PCI [16]	66.1±8.4	72.6	0	100.0	14.4	41.8	88.9	27.4
ETAMI [17]	N/A	72.6	100.0	87.1	74.2	19.4	54.8	8.1

N/A: not available, DISPERSE-2: dose confirmation study assessing anti-platelet effects of AZD6140 vs. clopidogrel in non-ST-segment elevation myocardial infarction-2, PLATO: platelet inhibition and patient outcomes, PHILO: ticagrelor vs. clopidogrel in Japanese, Korean, and Taiwanese patients with acute coronary syndrome, JUMBO-TIMI26: joint utilization of medications to block platelets optimally-thrombolysis in myocardial infarction 26, TRITON-TIMI 38: trial to assess improvement in therapeutic outcomes by optimizing platelet inhibition with prasugrel–thrombolysis in myocardial infarction, TRILOGY ACS: the targeted platelet inhibition to clarify the optimal strategy to medically manage acute coronary syndromes, TRIGGER PCI: testing platelet reactivity in patients undergoing elective stent placement on clopidogrel to guide alternative therapy with prasugrel, ETAMI: early thienopyridine treatment to improve primary PCI in patients with acute myocardial infarction ACS: acute coronary syndrome, PCI: percutaneous coronary intervention, MI: myocardial infarction.

*∗*Age is presented as either mean ± standard deviation or median (interquartile range) when available.

**Table 3:** Outcomes of the potent P2Y_12_ inhibitors.

Study	Heart failure		Cardiogenic Shock		Ventricular tachycardia		Ventricular fibrillation	
	Potent P2Y_12_ inhibitors	Clopidogrel	Potent P2Y_12_ inhibitors	Clopidogrel	Potent P2Y_12_ inhibitors	Clopidogrel	Potent P2Y_12_ inhibitors	Clopidogrel
DISPERSE-2 [11]*∗*	N/A	N/A	N/A	N/A	166/663	93/327	N/A	N/A
PLATO [1, 18]	196/9333	198/9291	60/9333	66/9291	29/9333	33/9291	48/9333	64/9291
PHILO [12, 19]	11/387	13/380	N/A	N/A	8/387	8/380	3/387	1/380
JUMBO-TIMI26 [13, 20]	1/651	0/254	N/A	N/A	N/A	N/A	N/A	N/A
TRITON-TIMI 38 [2, 21]	71/6741	92/6716	13/6741	8/6716	24/6741	25/6716	18/6741	25/6716
Ge et al. [14, 22]	2/463	4/229	4/463	2/229	3/463	1/229	1/463	0/229
TRILOGY ACS [15, 23]	199/4623	238/4617	15/4623	23/4617	15/4623	20/4617	3/4623	15/4617
TRIGGER PCI [16, 24]	1/210	5/210	N/A	N/A	0/210	1/210	0/210	0/210
ETAMI [17]	N/A	N/A	1/31	2/31	N/A	N/A	N/A	N/A

Values are n/N

N/A: not available, DISPERSE-2: dose confirmation study assessing anti-platelet effects of AZD6140 vs. clopidogrel in non-ST-segment elevation myocardial infarction-2, PLATO: platelet inhibition and patient outcomes, PHILO: ticagrelor vs. clopidogrel in Japanese, Korean, and Taiwanese patients with acute coronary syndrome, JUMBO-TIMI26: joint utilization of medications to block platelets optimally-thrombolysis in myocardial infarction 26, TRITON-TIMI 38: trial to assess improvement in therapeutic outcomes by optimizing platelet inhibition with prasugrel–thrombolysis in myocardial infarction, TRILOGY ACS: the targeted platelet inhibition to clarify the optimal strategy to medically manage acute coronary syndromes, TRIGGER PCI: testing platelet reactivity in patients undergoing elective stent placement on clopidogrel to guide alternative therapy with prasugrel, ETAMI: early thienopyridine treatment to improve primary PCI in patients with acute myocardial infarction.

*∗*Heart failure was defined as any reported congestive heart failure, acute and chronic heart failure.

†Ventricular tachycardia (VT) was categorized into sustained VT (lasting >30 s), non-sustained ventricular tachycardia (NSVT) (≥4 beats and <30 s in length), and triplets (3 ventricular beats).

## Data Availability

The outcome data used to support the findings of this study are included within the article in Table 3.

## Supplementary Materials

Supplementary Materials Supplementary Figure 1 showed the risk of bias of the included studies. Supplementary Figure 2 showed the funnel diagrams of the included studies. Supplementary Figure 3 showed forest the plots for pooled data of ventricular arrhythmias (studies with sample size>5000). Supplementary Figure 4 showed the forest plot for pooled data of cardiac dysfunction (studies with sample size>5000). Supplementary Figure 5 showed the forest plot for pooled data of ventricular arrhythmias (studies with time frame>6 months). Supplementary Figure 6 showed the forest plot for pooled data of cardiac dysfunction (studies with time frame>6 months).

## References

[1] Wallentin L., Becker R. C., Budaj A. (2009). Ticagrelor versus clopidogrel in patients with acute coronary syndromes. *The New England Journal of Medicine*.

[2] Wiviott S. D., Braunwald E., McCabe C. H. (2007). Prasugrel versus clopidogrel in patients with acute coronary syndromes. *The New England Journal of Medicine*.

[3] Valgimigli M., Bueno H., Byrne R. A. (2018). 2017 ESC focused update on dual antiplatelet therapy in coronary artery disease developed in collaboration with EACTS. *European Heart Journal*.

[4] Shah R., Rashid A., Hwang I., Fan T. M., Khouzam R. N., Reed G. L. (2017). Meta-Analysis of the Relative Efficacy and Safety of Oral P2Y12 Inhibitors in Patients With Acute Coronary Syndrome. *American Journal of Cardiology*.

[5] O'Gara P. T., Kushner F. G., Ascheim D. D. (2013). 2013 ACCF/AHA guideline for the management of st-elevation myocardial infarction: a report of the American College of Cardiology Foundation/American Heart Association Task Force on Practice Guidelines. *Journal of the American College of Cardiology*.

[6] Ibanez B., James S., Agewall S. (2018). 2017 ESC Guidelines for the management of acute myocardial infarction in patients presenting with ST-segment elevation. *European Heart Journal*.

[7] Roffi M., Patrono C., Collet J.-P. (2015). 2015 ESC guidelines for the management of acute coronary syndromes in patients presenting without persistent ST-segment elevation. *European Heart Journal*.

[8] Levine G. N., Bates E. R., Bittl J. A. (2016). 2016 ACC/AHA Guideline Focused Update on Duration of Dual Antiplatelet Therapy in Patients With Coronary Artery Disease: A Report of the American College of Cardiology/American Heart Association Task Force on Clinical Practice Guidelines: An Update of the 2011 ACCF/AHA/SCAI Guideline for Percutaneous Coronary Intervention, 2011 ACCF/AHA Guideline for Coronary Artery Bypass Graft Surgery, 2012 ACC/AHA/ACP/AATS/PCNA/SCAI/STS Guideline for the Diagnosis and Management of Patients With Stable Ischemic Heart Disease, 2013 ACCF/AHA Guideline for the Management of ST-Elevation Myocardial Infarction, 2014 AHA/ACC Guideline for the Management of Patients With Non–ST-Elevation Acute Coronary Syndromes, and 2014 ACC/AHA Guideline on Perioperative Cardiovascular Evaluation and Management of Patients Undergoing Noncardiac Surgery. *Circulation*.

[9] Priori S. G., Blomström-Lundqvist C., Mazzanti A. (2015). 2015 ESC Guidelines for the management of patients with ventricular arrhythmias and the prevention of sudden cardiac death. *European Heart Journal*.

[10] Dries D. L., Domanski M. J., Waclawiw M. A., Gersh B. J. (1997). Effect of antithrombotic therapy on risk of sudden coronary death in patients with congestive heart failure. *American Journal of Cardiology*.

[11] Storey R. F., Husted S., Harrington R. A. (2007). Inhibition of platelet aggregation by AZD6140, a reversible oral P2Y_12_ receptor antagonist, compared with clopidogrel in patients with acute coronary syndromes. *Journal of the American College of Cardiology*.

[12] Goto S., Huang C., Park S., Emanuelsson H., Kimura T. (2015). Ticagrelor vs. Clopidogrel in Japanese, Korean and Taiwanese Patients With Acute Coronary Syndrome– Randomized, Double-Blind, Phase III PHILO Study –. *Circulation Journal*.

[13] Wiviott S. D., Antman E. M., Winters K. J. (2005). Randomized comparison of prasugrel (CS-747, LY640315), a novel thienopyridine P2Y12 antagonist, with clopidogrel in percutaneous coronary intervention: results of the Joint Utilization of Medications to Block Platelets Optimally (JUMBO)-TIMI 26 trial. *Circulation*.

[14] Ge J., Zhu J., Hong B. (2010). Prasugrel versus clopidogrel in Asian patients with acute coronary syndromes: design and rationale of a multi-dose, pharmacodynamic, phase 3 clinical trial. *Current Medical Research and Opinion*.

[15] Roe M. T., Armstrong P. W., Fox K. A. A. (2012). Prasugrel versus clopidogrel for acute coronary syndromes without revascularization. *The New England Journal of Medicine*.

[16] Trenk D., Stone G. W., Gawaz M. (2012). A randomized trial of prasugrel versus clopidogrel in patients with high platelet reactivity on clopidogrel after elective percutaneous coronary intervention with implantation of drug-eluting stents: results of the TRIGGER-PCI (Testing Platelet Reactivity In Patients Undergoing Elective Stent Placement on Clopidogrel to Guide Alternative Therapy With Prasugrel) study. *Journal of the American College of Cardiology*.

[17] Zeymer U., Mochmann H., Mark B. (2015). Double-Blind, Randomized, Prospective Comparison of Loading Doses of 600 mg Clopidogrel Versus 60 mg Prasugrel in Patients With Acute ST-Segment Elevation Myocardial Infarction Scheduled for Primary Percutaneous Intervention. *JACC: Cardiovascular Interventions*.

[18] https://clinicaltrials.gov/ct2/show/results/NCT00391872?term=NCT00391872&amp;rank=1&amp;sect=X430156#othr

[19] https://clinicaltrials.gov/ct2/show/results/NCT01294462?term=NCT01294462&amp;rank=1&amp;sect=X430156#othr

[20] https://clinicaltrials.gov/ct2/show/results/NCT00059215?term=NCT00059215&amp;rank=1&amp;sect=X430156#othr

[21] https://clinicaltrials.gov/ct2/show/results/NCT00097591?term=NCT00097591&amp;rank=1&amp;sect=X430156#othr

[22] https://clinicaltrials.gov/ct2/show/results/NCT00830960?term=NCT00830960&amp;rank=1&amp;sect=X430156#othr

[23] https://clinicaltrials.gov/ct2/show/results/NCT00699998?term=NCT00699998&amp;rank=1&amp;sect=X30156#evnt

[24] https://clinicaltrials.gov/ct2/show/results/NCT00910299?term=NCT00910299&amp;rank=1&amp;sect=X430156#othr

[25] Moher D., Liberati A., Tetzlaff J., Altman D. G. (2009). Preferred reporting items for systematic reviews and meta-analyses: the PRISMA statement. *PLoS Medicine*.

[26] Collaboration TC Cochrane Handbook for Systematic Reviews of Interventions.

[27] Gorenek B., Blomström Lundqvist C., Brugada Terradellas J. (2014). Cardiac arrhythmias in acute coronary syndromes: position paper from the joint EHRA, ACCA, and EAPCI task force. *EP Europace*.

[28] Janse M. J., Wit A. L. (1989). Electrophysiological mechanisms of ventricular arrhythmias resulting from myocardial ischemia and infarction. *Physiological Reviews*.

[29] Pogwizd S. M., Corr P. B. (1987). Electrophysiologic mechanisms underlying arrhythmias due to reperfusion of ischemic myocardium. *Circulation*.

[30] Rossini R., Senni M., Musumeci G., Ferrazzi P., Gavazzi A. (2010). Prevention of left ventricular remodelling after acute myocardial infarction: An update. *Recent Patents on Cardiovascular Drug Discovery*.

[31] Pachel C., Mathes D., Arias-Loza A. (2016). Inhibition of Platelet GPVI Protects Against Myocardial Ischemia–Reperfusion Injury. *Arteriosclerosis, Thrombosis, and Vascular Biology*.

[32] Jia L., Qi G., Liu O. (2013). Inhibition of Platelet Activation by Clopidogrel Prevents Hypertension-Induced Cardiac Inflammation and Fibrosis. *Cardiovascular Drugs and Therapy*.

[33] ISIS-2 (Second International Study of Infarct Survival) Collaborative Group (1988). Randomised trial of intravenous streptokinase, oral aspirin, both, or neither among 17,187 cases of suspected acute myocardial infarction: ISIS-2. *The Lancet*.

[34] Yusuf S., Zhao F., Mehta S. R., Chrolavicius S., Tognoni G., Fox K. K. (2001). Effects of clopidogrel in addition to aspirin in patients with acute coronary syndromes without ST-segment elevation. *The New England Journal of Medicine*.

[35] Gurbel P. A., Bliden K. P., Hiatt B. L., O'Connor C. M. (2003). Clopidogrel for coronary stenting: response variability, drug resistance, and the effect of pretreatment platelet reactivity. *Circulation*.

[36] Bertling A., Fender A. C., Schüngel L. (2018). Reversibility of platelet P2Y12 inhibition by platelet supplementation:. *Journal of Thrombosis and Haemostasis*.

[37] Marsousi N., Daali Y., Fontana P. (2018). Impact of Boosted Antiretroviral Therapy on the Pharmacokinetics and Efficacy of Clopidogrel and Prasugrel Active Metabolites. *Clinical Pharmacokinetics*.

[38] Rafique A. M., Nayyar P., Wang T. Y. (2016). Optimal P2Y 12 Inhibitor in Patients With ST-Segment Elevation Myocardial Infarction Undergoing Primary Percutaneous Coronary Intervention. *JACC: Cardiovascular Interventions*.

[39] Sahlén A., Varenhorst C., Lagerqvist B. (2016). Outcomes in patients treated with ticagrelor or clopidogrel after acute myocardial infarction: experiences from SWEDEHEART registry. *European Heart Journal*.

[40] Khan J. N., Greenwood J. P., Nazir S. A. (2016). Infarct Size Following Treatment With Second‐ Versus Third‐Generation P2Y. *Journal of the American Heart Association*.

[41] Lip G., Ponikowski P., Andreotti F. (2017). Thromboembolism and antithrombotic therapy for heart failure in sinus rhythm. *Thrombosis and Haemostasis*.

[42] Wittfeldt A., Emanuelsson H., Brandrup-Wognsen G. (2013). Ticagrelor Enhances Adenosine-Induced Coronary Vasodilatory Responses in Humans. *Journal of the American College of Cardiology*.

[43] Nylander S., Femia E., Scavone M. (2013). Ticagrelor inhibits human platelet aggregation via adenosine in addition to P2Y. *Journal of Thrombosis and Haemostasis*.

[44] Sumaya W., Storey R. F. (2017). Ticagrelor. *Interventional Cardiology Clinics*.

[45] Nanhwan M. K., Ling S., Kodakandla M., Nylander S., Ye Y., Birnbaum Y. (2014). Chronic Treatment With Ticagrelor Limits Myocardial Infarct Size. *Arteriosclerosis, Thrombosis, and Vascular Biology*.

[46] Ye Y., Birnbaum G. D., Perez-Polo J. R., Nanhwan M. K., Nylander S., Birnbaum Y. (2015). Ticagrelor Protects the Heart Against Reperfusion Injury and Improves Remodeling After Myocardial Infarction. *Arteriosclerosis, Thrombosis, and Vascular Biology*.

[47] Liverani E., Rico M. C., Garcia A. E., Kilpatrick L. E., Kunapuli S. P. (2012). Prasugrel Metabolites Inhibit Neutrophil Functions. *The Journal of Pharmacology and Experimental Therapeutics*.

[48] Watti H., Dahal K., Zabher H. G., Katikaneni P., Modi K., Abdulbaki A. (2017). Comparison of prasugrel and ticagrelor in patients with acute coronary syndrome undergoing percutaneous coronary intervention: A meta-analysis of randomized and non-randomized studies. *International Journal of Cardiology*.

