# Supplementary material for: Effect of Potent P2Y12 Inhibitors on Ventricular Arrhythmias and Cardiac Dysfunction in Coronary Artery Disease: A Systematic Review and Meta-Analysis
Source: Biomed Res Int. 2018 Dec 17;2018:8572740. doi: 10.1155/2018/8572740 (PMC6311824; doi:10.1155/2018/8572740)
Supplement: Supplementary Materials — Supplementary Figure 1 showed the risk of bias of the included studies. Supplementary Figure 2 showed the funnel diagrams of the included studies. Supplementary Figure 3 showed forest the plots for pooled data of ventricular arrhythmias (studies with sample size>5000). Supplementary Figure 4 showed the forest plot for pooled data of cardiac dysfunction (studies with sample size>5000). Supplementary Figure 5 showed the forest plot for pooled data of ventricular arrhythmias (studies with time frame>6 months). Supplementary Figure 6 showed the forest plot for pooled data of cardiac dysfunction (studies with time frame>6 months). [file 8572740.f1.pdf]

Supplementary Data

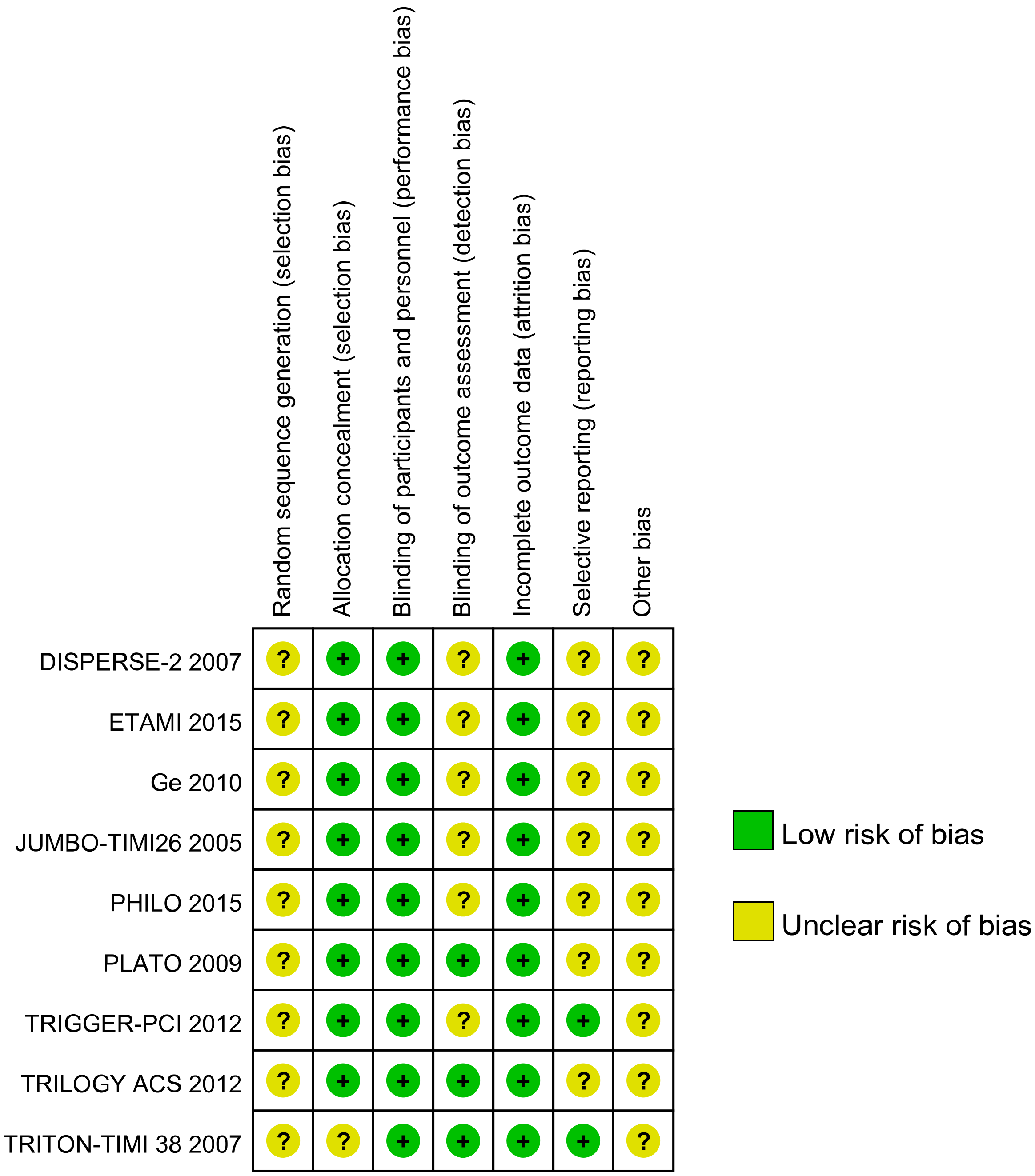

Supplementary Fig. 1. Risk of bias of the included studies.

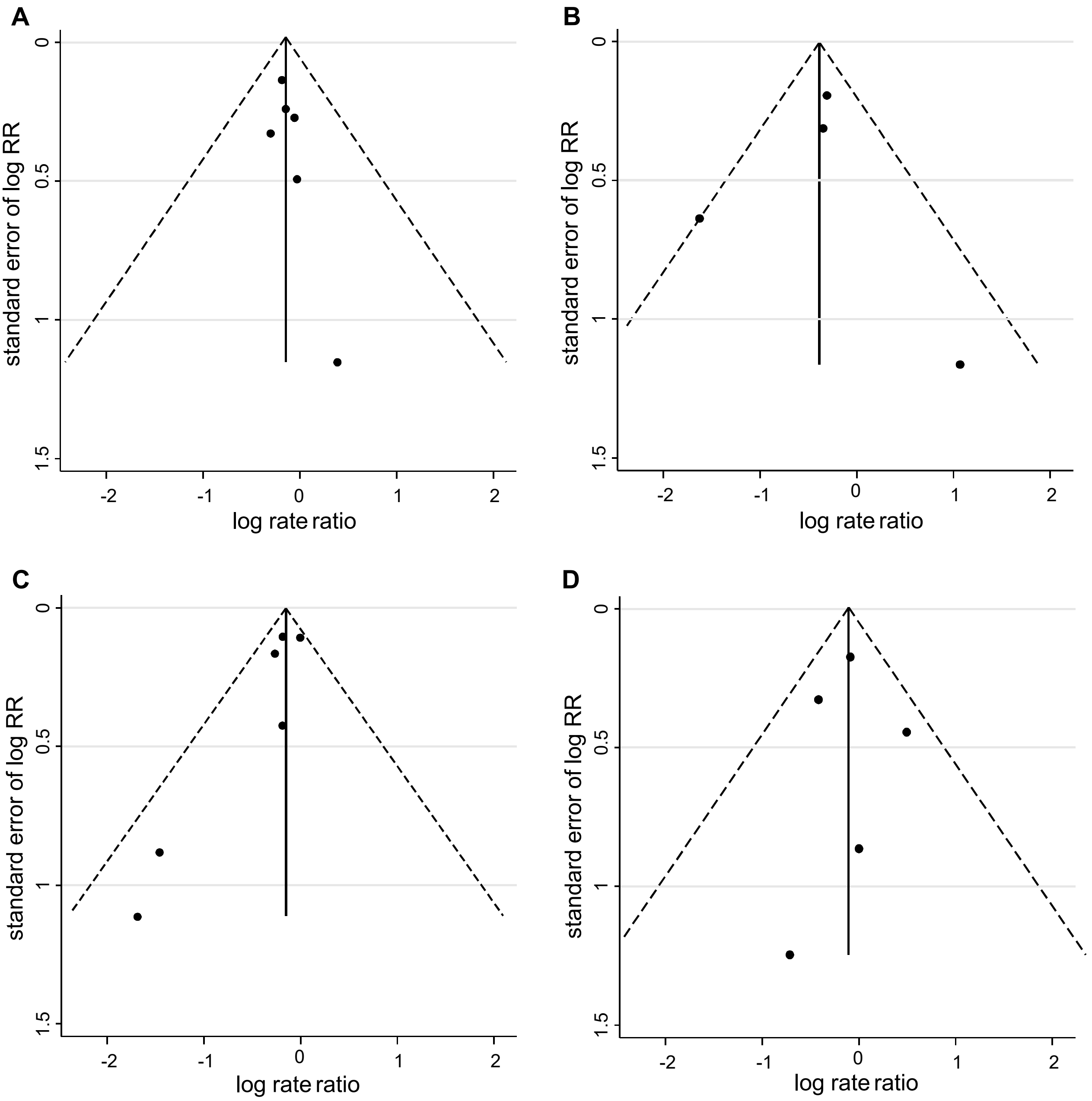

Supplementary Fig. 2. Funnel diagrams of the included Studies. A, funnel diagrams for ventricular tachycardia; B, funnel diagrams for ventricular fibrillation; C, funnel diagrams for heart failure; D, funnel diagrams for cardiogenic shock.

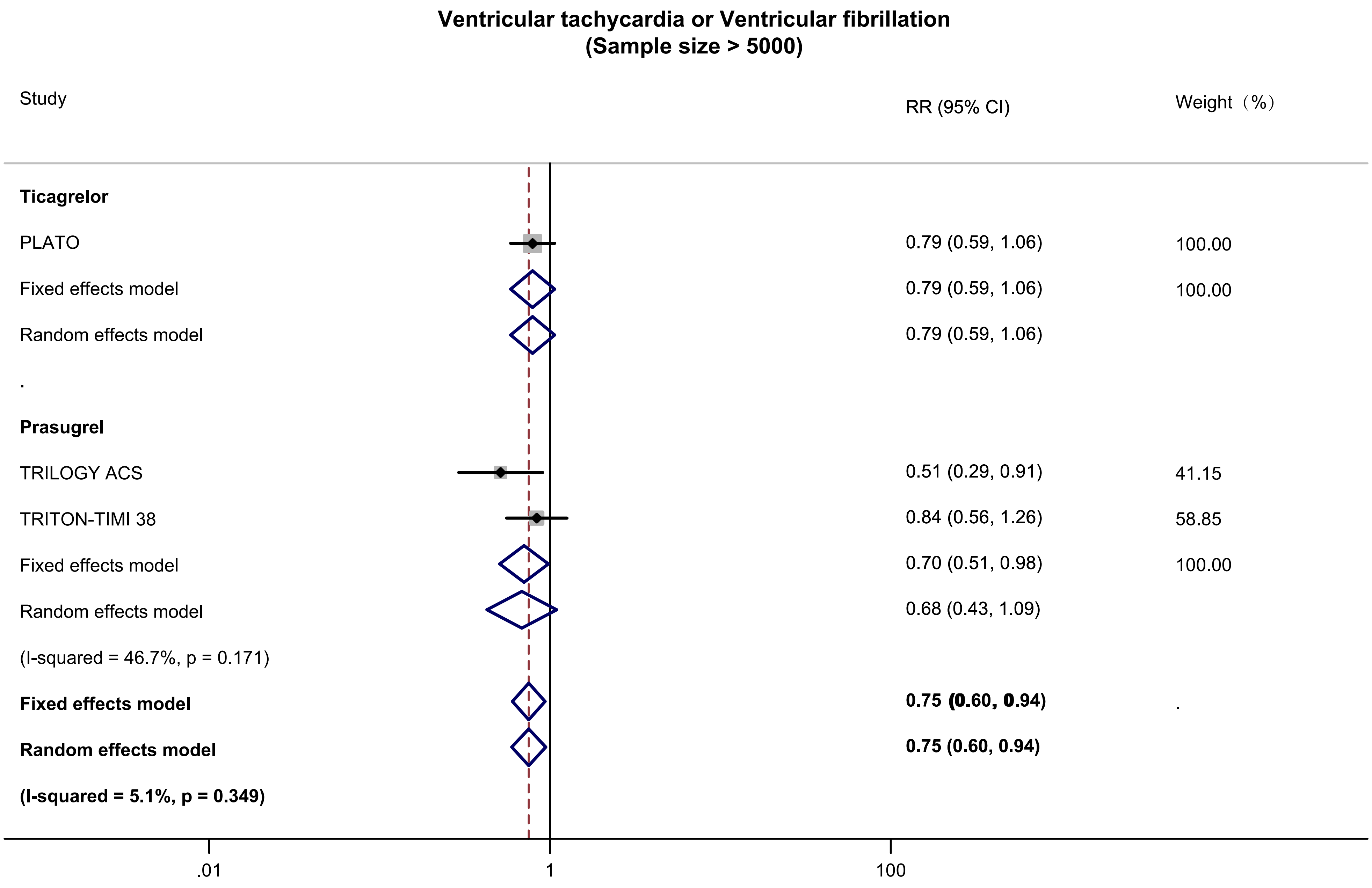

Supplementary Fig. 3. Forest plot for pooled data of ventricular arrhythmias (Studies with sample size>5000).

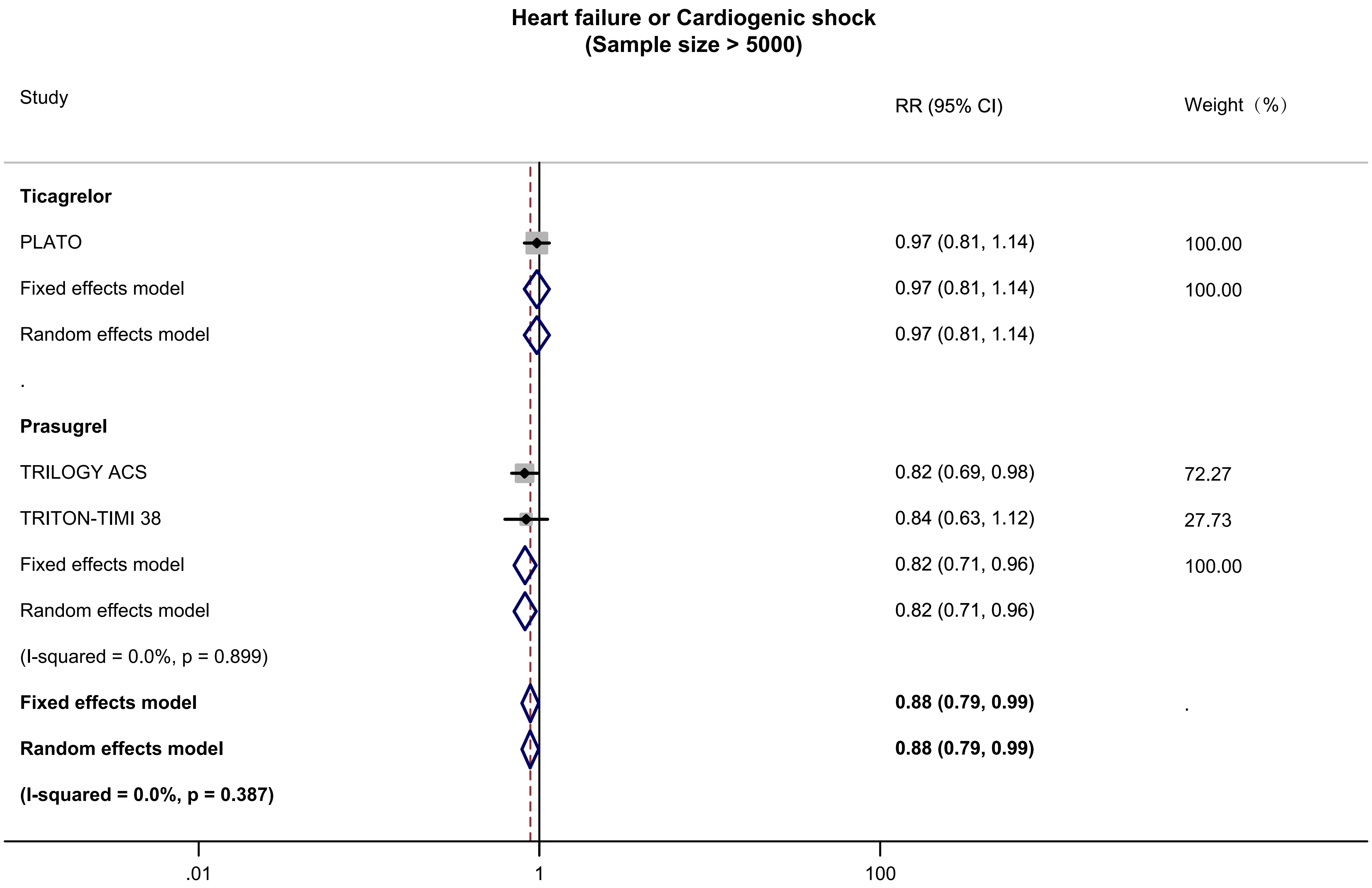

Supplementary Fig. 4. Forest plot for pooled data of cardiac dysfunction (Studies with sample size>5000).

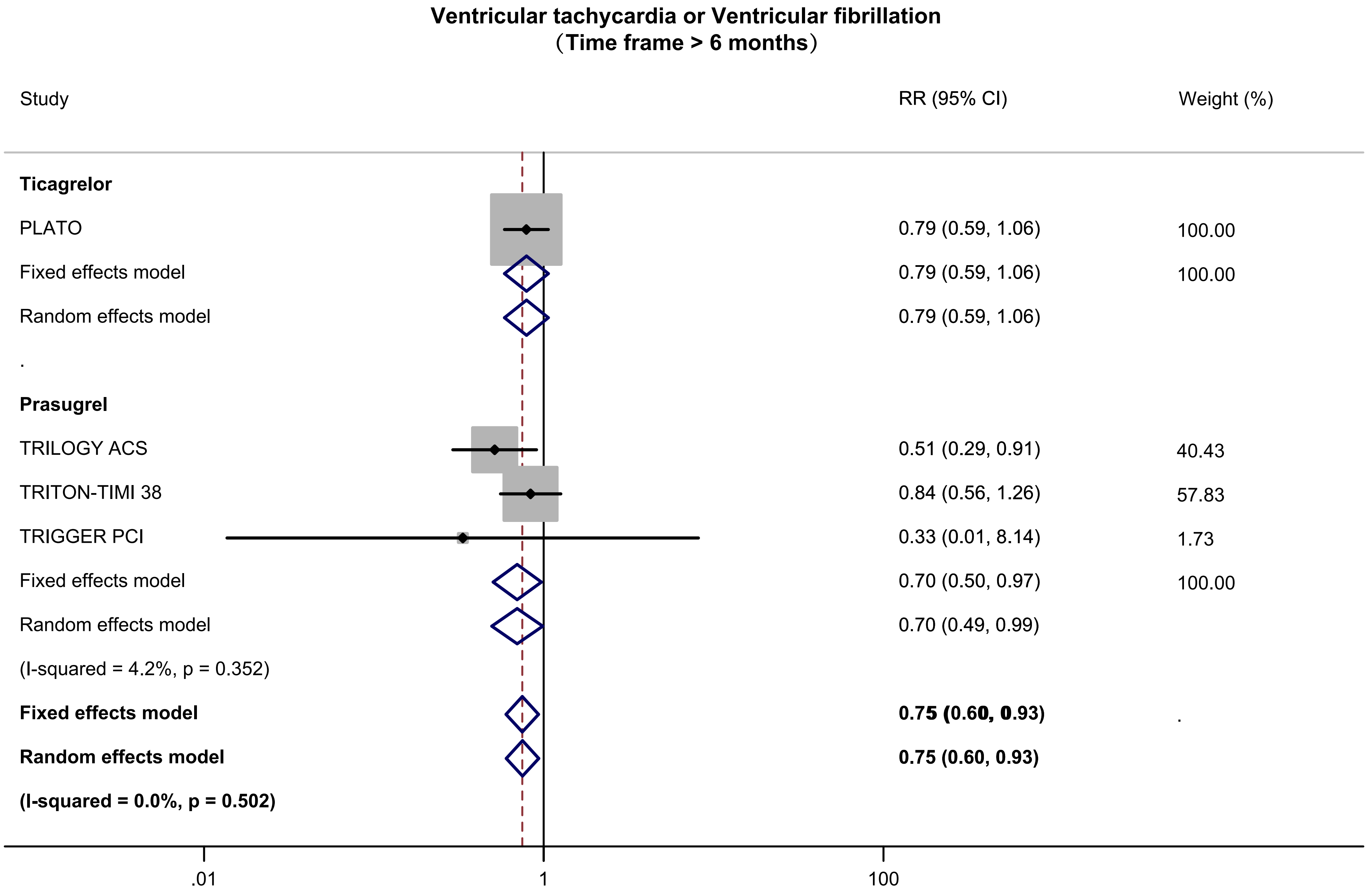

Supplementary Fig. 5. Forest plot for pooled data of ventricular arrhythmias (Studies with time frame>6 months).

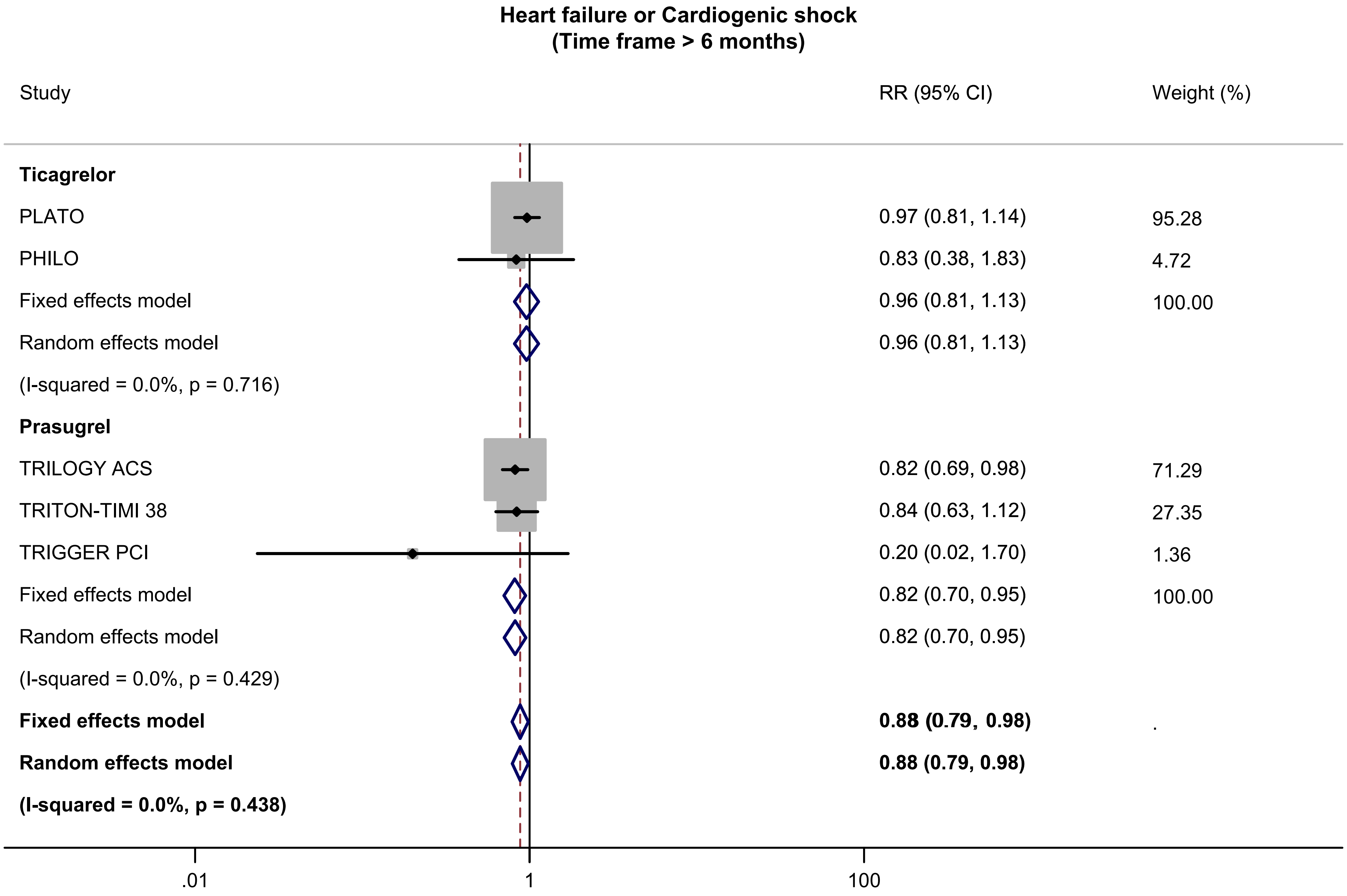

Supplementary Fig. 6. Forest plot for pooled data of cardiac dysfunction (Studies with time frame>6 months).
